# Structures of annexin A2-PS DNA complexes show dominance of hydrophobic interactions in phosphorothioate binding

**DOI:** 10.1093/nar/gkac774

**Published:** 2022-09-17

**Authors:** Malwina Hyjek-Składanowska, Brooke A Anderson, Vitaliy Mykhaylyk, Christian Orr, Armin Wagner, Jarosław T Poznański, Krzysztof Skowronek, Punit Seth, Marcin Nowotny

**Affiliations:** Laboratory of Protein Structure, International Institute of Molecular and Cell Biology, Warsaw 02-109, Poland; Ionis Pharmaceuticals, Inc., Carlsbad, CA 92010, USA; Diamond Light Source, Harwell Campus, Didcot OX11 0DE, UK; Diamond Light Source, Harwell Campus, Didcot OX11 0DE, UK; Diamond Light Source, Harwell Campus, Didcot OX11 0DE, UK; Institute of Biochemistry and Biophysics, Polish Academy of Sciences, Warsaw, Poland; Biophysics and Structural Biology Facility, International Institute of Molecular and Cell Biology, Warsaw, Poland; Ionis Pharmaceuticals, Inc., Carlsbad, CA 92010, USA; Laboratory of Protein Structure, International Institute of Molecular and Cell Biology, Warsaw 02-109, Poland

## Abstract

The introduction of phosphorothioate (PS) linkages to the backbone of therapeutic nucleic acids substantially increases their stability and potency. It also affects their interactions with cellular proteins, but the molecular mechanisms that underlie this effect are poorly understood. Here, we report structural and biochemical studies of interactions between annexin A2, a protein that does not possess any known canonical DNA binding domains, and phosphorothioate-modified antisense oligonucleotides. We show that a unique mode of hydrophobic interactions between a sulfur atom of the phosphorothioate group and lysine and arginine residues account for the enhanced affinity of modified nucleic acid for the protein. Our results demonstrate that this mechanism of interaction is observed not only for nucleic acid-binding proteins but can also account for the association of PS oligonucleotides with other proteins. Using the anomalous diffraction of sulfur, we showed that preference for phosphorothioate stereoisomers is determined by the hydrophobic environment around the PS linkage that comes not only from protein but also from additional structural features within the ASO such as 5-Me groups on cytosine nucleobases.

## INTRODUCTION

The phosphorothioate (PS) backbone is a commonly used component of therapeutic nucleic acids (1). The PS linkage protects single- and double-stranded oligonucleotide drugs from nucleolytic cleavage, thereby enhancing tissue half-life and pharmacological activity. Additionally, extensive PS modifications of single-stranded antisense oligonucleotides (ASOs) enhance their affinity for plasma, cell-surface, and intracellular proteins, facilitating cellular uptake and tissue distribution (2). For example, Spinraza™ is a fully PS 2′-methoxyethyl (MOE)-modified ASO that is approved for the treatment of all forms of spinal muscular atrophy (SMA) (3).

Over the past decade, significant progress has been made toward identifying cellular proteins that interact with PS ASOs (2). The strength of protein-ASO interactions is determined by the total number of PS linkages, strand flexibility, and additional modifications that are used to modulate drug-like properties of ASO therapeutics (4). Overall, protein binding increases with PS content, suggesting an avidity model whereby each PS contributes a small percentage to total binding energy. The inherent flexibility of single-stranded ASOs presumably allows interactions with different regions of the protein surface. Other chemical modifications, such as 2′-fluoro (2′-F), MOE and 2′-constrained ethyl (cEt) further modulate these interactions by altering local conformation, steric restraints, and hydrophobicity in vicinity of the PS backbone.

As part of a comprehensive effort to understand molecular mechanisms of PS ASO-protein interactions, we recently reported the co-crystal structure of a PS ASO that is bound to PC4, a transcription factor that was shown to affect ASO activity (5). We showed that the PS linkage can form simultaneous electrostatic and hydrophobic interactions with arginine and lysine side chains. In the present study, we extended these observations to another ASO-interacting protein, annexin A2 (AnxA2).

Annexin A2 is a non-EF-hand calcium-binding protein that is characterized by the ability to bind and aggregate (i.e. ‘annex’) anionic phospholipid membranes (6). Apart from its role in vesicular transport, exocytosis and endocytosis, this multifunctional protein is also involved in signal transduction and regulation of membrane-cytoskeleton contacts (7,8). AnxA2 preferentially binds to phospholipids and other anionic polymers and does not contain any canonical RNA- or DNA-binding motifs. Several studies, however, reported functional calcium-dependent interactions between this protein and both coding (9,10) and long-noncoding RNAs (11). The detailed mechanisms of these interactions are not well understood. Importantly, AnxA2 was shown to bind phosphorothioate oligonucleotides (12) and has been implicated in the release of PS ASOs from endo-lysosomal compartments in productive ASO cellular trafficking (13).

In the present study, we co-crystallized the C-terminal domain (CTD) core of AnxA2 with a PS 2′-MOE DNA gapmer ASO and found that unique hydrophobic interactions between PS groups and lysine and arginine residues account for the enhanced affinity of PS ASO to the protein surface. Our results demonstrate that this mechanism of interaction is a more general phenomenon that is observed not only for nucleic acid-binding proteins but may also rationalize ASO association with other classes of proteins.

## MATERIALS AND METHODS

### Protein expression and purification

The synthetic gene for full-length human AnxA2 was obtained from OriGene Technologies, USA. The fragment corresponding to the C-terminal domain of AnxA2 (CTD; residues 34-339) was subcloned into the pET28_HisSUMOTag expression vector between BamHI/XhoI cloning sites. All AnxA2 variants with point substitutions were obtained as synthetic genes from Biomatik (Ontario, Canada), cloned into the pET28-HisSUMO vector. All proteins (wildtype and mutants) were expressed in the *Escherichia coli* BL21(DE3) strain. The cultures were grown at 37°C for 72 h in Auto Induction Medium (AIM) Super Broth Base, including trace elements (Formedium, UK), which contained 1% glycerol and 30 μg/ml kanamycin. The cells were harvested by centrifugation. Pellets were washed with phosphate-buffered saline (PBS), frozen in liquid nitrogen, and stored at −20°C. For purification, the pellets were resuspended in lysis buffer (20 mM HEPES [pH 7.0], 150 mM NaCl, 5 mM β-mercaptoethanol [β-Me], 20 mM imidazole, 1 mM phenylmethylsulfonyl fluoride, 307 nM aprotinin, 1 μM pepstatin A, and 10 μM leupeptin) and disrupted via sonication. Following centrifugation at 30 000 rpm at 4°C, the cleared lysate was loaded onto a HisTrap FF crude column (GE Healthcare) that was equilibrated with 20 mM HEPES (pH 7.0), 150 mM NaCl, 5 mM β-Me and 20 mM imidazole. After washing with equilibration buffer, the proteins were eluted with 20 mM HEPES (pH 7.0), 150 mM NaCl, 5 mM β-Me, and 300 mM imidazole. The eluted fractions were subjected to SUMO protease digestion and dialyzed against 20 mM HEPES (pH 7.0), 150 mM NaCl, 5 mM β-Me, and 20 mM imidazole. The proteins were next reapplied onto a HisTrap column (GE Healthcare). The flow-through and wash fractions that contained tag-less protein were pooled, concentrated, and subjected to size-exclusion chromatography on a 16/600 Superdex 75 pg column (GE Healthcare) that was equilibrated with 20 mM HEPES (pH 7.0), 100 mM NaCl and 1 mM dithiothreitol. Peak fractions that contained purified AnxA2 were pooled and concentrated to 20 mg/ml. Protein aliquots were flash-frozen in liquid nitrogen and stored at −80°C.

### Crystallization

Purified AnxA2 protein was mixed with 5–10 PS MOE DNA gapmer MALAT ASO (Ionis# 1590111) at a 1:1 molar ratio to a final protein concentration of 9 mg/ml and incubated on ice for 30 min. Crystallization trials were performed at 18°C using the sitting-drop vapor diffusion method. Initial crystallization hits were identified in Morpheus Screen (Molecular Dimensions, USA). After the optimization of crystal growth conditions, the best diffracting crystals were obtained for the AnxA2-ASO complex mixed with an equal volume of reservoir buffer that contained 4% w/v PEG 4000, 14% v/v glycerol, 0.03 M MgCl_2_/CaCl_2_, and 0.1 M bicine/Trizma base (pH 8.3; for structure I) or 12% w/v PEG 8000, 24% v/v ethylene glycol, 0.02 M sodium formate/ammonium acetate/trisodium citrate/sodium potassium L-tartrate/sodium oxamate, and 0.1 M MES/imidazole (pH 6.1; for structure II) using the hanging-drop vapor diffusion method. Crystals were cryo-protected in 30% glycerol (for structure I) or 20% ethylene glycol (for structure II) and flash frozen in liquid nitrogen.

### Structure determination and refinement

X-ray diffraction data for structure I were collected at the PETRA III storage ring at the P13 beamline, operated by EMBL Hamburg (14), at a wavelength of λ = 0.9763 Å. The crystals diffracted to a maximum resolution of 1.87 Å. The dataset was next processed and scaled using XDS (15). Phases were determined using molecular replacement in the Phaser-MR module of Phenix (16). The structure of human AnxA2 (PDB ID: 5LPU, chain A) was used as the search model. DNA was built manually in Coot (17,18). To determine the position of non-bridging sulfur atoms in stereorandom PS ASO oligonucleotides, data were collected at long‐wavelength beamline I23 at Diamond Light Source (19) at a wavelength of λ = 2.7552 Å. This wavelength was used because it provides a good balance between increased anomalous signal from sulfur and increased X-ray absorption. Each dataset consisted of 360° with an exposure time of 0.1 s per 0.1° image. Multiple datasets per crystal were taken at varying kappa and phi values to ensure completeness and increase multiplicity. Data integration was performed with XDS and XSCALE (15). The origin of the PDB model was corrected using POINTLESS, AIMLESS, and MOLREP (20,21). Phased anomalous difference Fourier maps were subsequently produced using ANODE (22). The calculated anomalous difference maps were next used to select phosphorothioate stereoisomers which were built into the structural model. For the phosphates for which the anomalous difference map clearly indicated the *R*p or *S*p PS configuration, single stereoisomer was built into the model. Nucleotides for which no anomalous electron densities were visible or for which the densities were evenly distributed at both sides of the phosphorus atoms were refined as two alternative conformations that represented *R*p and *S*p PS diastereoisomers, and each stereoisomer was refined at 0.5 occupancy.

Diffraction data for structure II were collected at the P11 DESY beamline (23) at a wavelength of λ = 1.0332 Å. The crystals diffracted X-rays to a maximum resolution of 2.4 Å. The dataset was processed and scaled using XDS (15). Phases were determined using molecular replacement in the Phaser-MR module of Phenix (16). The structure of human AnxA2 (PDB ID: 5LPU, chain A) was used as the search model. DNA was built manually in Coot. Because of the low quality of electron density maps for nucleobases, it was not possible to establish the register of the ASO; therefore, an arbitrarily chosen dT sequence was built in the model. Discontinuous electron density that corresponded to the remaining part of the oligonucleotide could also be observed, although it was not sufficiently clear to be modeled with confidence. Each nucleotide was refined as two alternative conformations that represented *R*p and *S*p PS diastereoisomers. Each stereoisomer was refined at 0.5 occupancy.

The models were subsequently improved by multiple rounds of manual building in Coot (17,18), followed by refinement using Phenix.refine, with *R*_free_ calculated with 5% unique reflections. The geometric restraints for modified nucleotides were generated using Global Phasing Grade Web server (24) and next corrected manually with the reference values from Parkinson *et al.* (25). The prepared .cif restraints files were next used for model building in Coot and refinement in Phenix.refine. Structure validation was performed using MolProbity (26). Data collection and refinement statistics are listed in Table 1. The structural analysis was performed using PyMOL, which was also used to prepare the figures (PyMOL Molecular Graphics System, version 2.3.3, Schrödinger, LLC).

**Table 1.** Crystallographic data and refinement statistics

	Structure I	Structure I anomalous data	Structure II
Data collection			
Wavelength (Å)	0.9763	2.7552	1.0332
Space group	*P*2_1_	*P*2_1_	*P*2_1_
Cell dimensions			
*a*, *b*, *c* (Å)	55.62, 57.50, 70.48	55.49, 57.57, 70.48	50.23, 60.36, 128.43
α, β, γ (°)	90.00, 90.24, 90.00	90.00, 90.15, 90.00	90.00, 97.17, 90.00
Resolution range (Å)	43.75–1.87 (1.99–1.87)^a^	70.51–2.10 (2.16–2.10)*	44.47–2.40 (2.54–2.40)*
Completeness (%)	99.8 (99.0)^a^	93.6 (86.1)*	96.5 (82.9)*
*R*_merge_ (%)	14.4 (129.5)^a^	10.9 (119.3)^a^	29.65 (178.4)^a^
*I*/σ(*I*)	12.1 (2.0)^a^	11.3 (1.6)^a^	9.0 (2.0)^a^
Redundancy	6.6 (5.6)^a^	9.5 (7.8)^a^	6.9 (6.2)^a^
CC1/2	1.0 (0.72)^a^	0.99 (0.60)^a^	0.99 (0.54)^a^
**Refinement**			
Resolution range (Å)	43.75–1.87 (1.94–1.87)^a^		35.85–2.40 (2.48–2.40)^a^
Unique reflections	36 718 (2010)^b^		29 132 (1456)^b^
*R*/*R*_free_ (%)	16.7/19.8		18.5/24.2
Number of atoms	3768		5445
Protein	2472		4841
Nucleic acid	888		300
Ion	8		11
Water	352		241
*B*-factors (Å^2^)	36.8		44.3
Protein	29.0		39.9
Nucleic acid	56.0		116.1
Ion	31.0		78.6
Water	42.3		42.4
rmsd			
Bond lengths (Å)	0.009		0.008
Bond angles (°)	1.000		0.950

The data collection statistics are based on a single crystal.

^a^Values in parentheses are for highest-resolution shell.

^b^Number of reflections used for *R*_free_ calculation in parentheses.

### Fluorescence anisotropy

The binding of PS and PO oligonucleotides to wildtype AnxA2 and its mutated variants was studied using fluorescence anisotropy. The assay was conducted in 96-well, black, flat-bottom polystyrene NBS plates (Corning, catalog no. 3991) in a total reaction volume of 60 μl. Fluorescently labeled oligonucleotides were used at a concentration of 50 nM, and the protein concentration ranged from 0 to 120 μM. Dilutions of the oligonucleotides and protein were prepared in buffer A (20 mM HEPES [pH 7.0], 100 mM NaCl, and 1 mM DTT). Depending on the experimental variant, the binding reactions were conducted in buffer A that contained 1 mM CaCl_2_, 1 mM MgCl_2_, or 1 mM EGTA. The reactions were prepared in triplicate. Fluorescence anisotropy was measured using a Tecan Infinite M1000 microplate reader at excitation/emission wavelengths of 530/568 nm for Cy3-labeled oligonucleotides and 635/670 nm for Alexa647-labeled oligonucleotides. The results of fluorescence anisotropy titrations were analyzed according to the 1:1 binding model. The standard equation was modified to describe the effect of unspecific noncompetitive protein–oligonucleotide interaction, which decreases an apparent population of the protein, ${{Prot}}{{{}}}_{{{total}}},$ by a factor ${\rm{NS}}*{{Prot}}{{{}}}_{{{total}}}{\rm{.}}$ The fitting was performed using of the following equation:

$$\begin{equation*}Anisotropy = {A}_0 + \frac{{\left( {\Delta A * \left( {Pro{t}_{RNA} + NS * Pro{t}_{total}} \right)} \right)}}{{RN{A}_{total}}}\end{equation*}$$

under the following constraints:

$$\begin{equation*} RN{A}_{total} = RN{A}_{free} + Pro{t}_{RNA} \end{equation*}$$

$$\begin{equation*} Pro{t}_{total}* \left( {1 - NS} \right) = Pro{t}_{free} + Pro{t}_{RNA} \end{equation*}$$

$$\begin{equation*} Pro{t}_{free}* RN{A}_{free} = {K}_D *Pro{t}_{RNA} \end{equation*}$$

where RNA_total_ and Prot_total_ are the total concentration of the labeled RNA and protein, respectively, RNA_free_ and Prot_free_ are concentrations of free species. Prot_RNA_ is the concentration of the specific 1:1 complex, and NS*Prot_total_ is the concentration of a nonspecific protein–RNA complex, formation of which does not interfere with the specific protein–RNA binding. We have assumed that specific and nonspecific binding similarly affect RNA fluorescence. The latter system of equations leads to the quadratic expression against Prot_RNA_, which could be explicitly resolved by roots.

For each system, three independent series of experiments were analyzed globally, assuming the common values of *K*_D_, spectral parameters (baseline, *A*_0_ and anisotropy change upon binding, Δ*A*), and NS. The hypothesis NS > 0 was always tested against the alternative NS = 0 according to Akaike's Information Criterion Test (AIC). All identified outliers were removed from the analysis (denoted in gray in Figures 4 and 5). The analyses were performed with Origin Pro (ver 9.9, www.originlab.com).

### Fourier-transform infrared spectroscopy

Secondary structure analysis for wildtype AnxA2 and its mutated variants was performed using a Bruker Tensor 27 FT-IR spectrometer. Protein samples were used at a concentration of 2 mg/ml in a buffer that contained 20 mM HEPES (pH 7.0), 100 mM NaCl and 1 mM DTT with and without 1 mM CaCl_2_. The spectra were analyzed using OPUS-QUANT2 software with the protein spectrum library that was provided by the supplier as a reference.

## RESULTS

### Crystallization of AnxA2–ASO complexes

**Figure 1.**
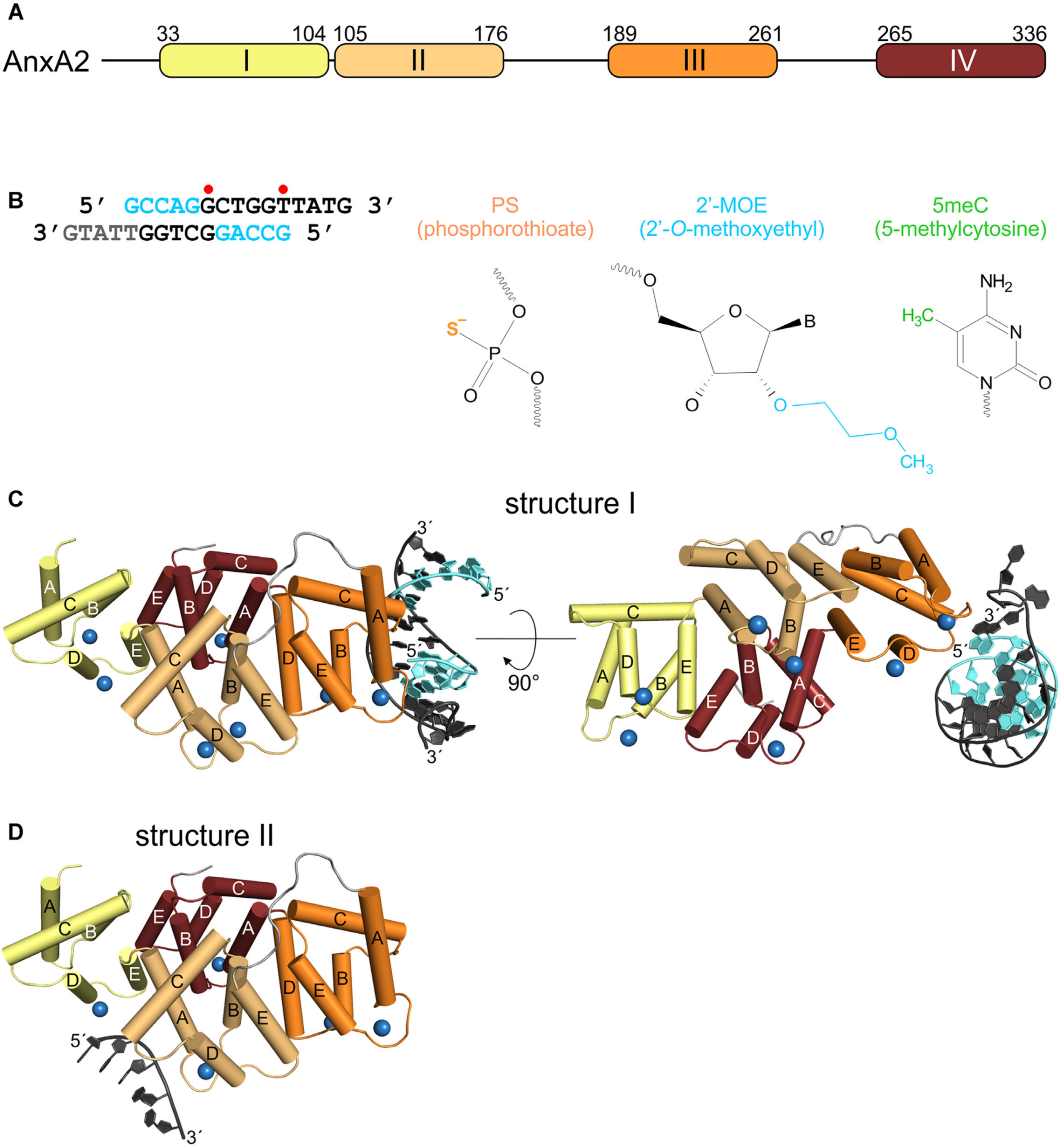
Structures of AnxA2-ASO complexes. (**A**) Schematic representation of domain composition of AnxA2. Calcium-binding annexin domains I–IV are colored yellow, pale orange, orange and ruby, respectively. The amino acid numbers at the boundaries of each annexin repeat are given. (**B**) Nucleotide sequence and chemical structure of MALAT PS ASO used in this study. The left panel shows the ASO sequence, presented in double-stranded form, observed in structure I. 2′-MOE PS nucleotides are shown in cyan, and PS DNA gapmer nucleotides are shown in black. Nucleotides that are not visible in the electron density map are shown in gray. Red dots indicate mismatches in the duplex. All cytidines in the ASO were 5-methylated. The right panel shows chemical structures of modified nucleotides that are present in the ASO. (**C**) Structure I. Annexin A2 is shown in cartoon representation, with Ca^2+^-binding annexin domains colored as in (A). The conserved α-helices A–E are indicated. 2′-MOE nucleotides are shown in cyan. DNA gapmer nucleotides are shown in dark gray. Ca^2+^ ions are shown as blue spheres. (**D**) Structure II shown as in (C).

To broaden our understanding of the mechanism of PS ASO binding to proteins, we sought to determine structures of fully phosphorothioate ASOs in complex with a nonspecific DNA binder, AnxA2 protein. Annexin A2 is composed of a flexible, 33-amino-acid N-terminal fragment and conserved CTD (Figure 1A). For our studies, we used a truncated variant of the protein that comprised the CTD core (amino acids 34–339), hereafter referred to as AnxA2. After extensive crystallization trials with several ASOs with different sequences, chemical compositions, and lengths, we obtained protein-nucleic acid co-crystals for a 5–10 PS 2′-MOE DNA gapmer ASO, with a sequence that targets human metastasis-associated lung adenocarcinoma transcript 1 (*MALAT*) long non-coding RNA. This ASO contained five nucleotides that are 2′-methoxyethyl RNA-modified, followed by 10 DNA nucleotides that comprise the ‘gap’ part of the ASO (Figure 1B). The phosphorothioate backbone was a racemic mixture of PS *R*p/*S*p diastereoisomers, which is typical for therapeutic oligonucleotides. Additionally, the cytosine bases were substituted with 5-methylcytosine (m^5^C), which is another modification that is widely employed in antisense therapeutics.

From our crystallization trials, we obtained two types of AnxA2–ASO co-crystals that were used to solve two structures referred to as structure I and structure II (Figure 1C, D), determined at 1.9 and 2.4 Å resolution, respectively. Both crystal types belonged to the *P*2_1_ space group but with different unit cell parameters and different crystal packing (Table 1). The asymmetric unit in structure I contains one AnxA2–ASO complex. In structure II, it contains two copies of the complex (complex 1 and complex 2). The protein chains in both structures have a virtually identical conformation and can be superimposed with a root-mean-square deviation (rmsd) of 0.4 Å over 296 C-α atoms. The major difference between the two structures is that the nucleic acid is bound by AnxaA2 using two different interfaces (Figure 1C, D). Additionally, the asymmetric unit of structure I contains two chains of partially base-paired ASO, which form a largely ordered duplex in the structure. As a result, the electron density for the oligonucleotide in structure I is much better defined, which allowed unambiguous tracing of the DNA (Supplementary Figure S1A). In structure II, only a small single-stranded part that comprised three or six (for complexes 2 and 1, respectively) of 15 nucleotides could be built in the model (Supplementary Figure S1B). In this crystal, the electron density indicated the presence of double-stranded nucleic acid that was formed by ASOs from complexes 1 and 2. However, the quality of the map only allowed the tracing of fragments of nucleic acids that were not base-paired. Nevertheless, both crystal types that we obtained contained ASO in duplex form. Because of low quality of the electron density maps for nucleobases, it was not possible to establish the register of the ASO in structure II; therefore, an arbitrarily chosen dT sequence was built in the model. In the description below, we focus on the better-defined structure I and, where relevant, compare it with structure II.

### Crystal structures of AnxA2–ASO complex reveal protein interactions with the phosphorothioate backbone

The comparison of structure I with the previously reported apo structure of AnxA2 (Protein Data Bank [PDB] ID: 1XJL) shows that ASO binding does not cause any significant conformational changes in the protein. The two structures are very similar and can be superimposed, with an rmsd of 0.4 Å over 289 C-α atoms. The protein forms a compact core structure and is shaped as a slightly curved disk (Figure 1C). The structure is divided into four highly homologous repeats (so-called annexin domains I–IV), arranged in a near parallel orientation. Each 70-amino-acid domain is composed of five α-helices, named A–E. The convex side of the protein participates in binding to anionic phospholipids and other negatively charged polymers and accommodates up to seven Ca^2+^ ions.

The asymmetric unit of structure I contains one AnxA2 protomer and two chains of ASO. For one DNA strand, all 15 nucleotides were clearly visible, whereas for the second strand, 10 nucleotides could be built in the model. Unexpectedly, partial complementarity of the ASO region that encompasses nucleotides 2–10 promoted intermolecular base pairing. As a result, two strands form a largely ordered duplex in the structure (Figure 1B, C). Most of the duplex shows canonical Watson-Crick base base-pairing. The complementary region contains two mismatches with an altered base-pair conformation. One irregularity is the G-G mismatch that is located at position 6 of both strands, in which guanine bases adopt alternative conformations with *syn-* and *anti*-configurations. This leads to the formation of non-canonical Hoogsteen G-G base pairing in two alternative conformations. The other irregularity is observed at dT11 of one of the strands, which does not base pair with the opposing strand. Instead, the nucleobase is flipped out from the duplex, and the phosphorothioate backbone adopts a distorted conformation.

The 5′ ends of the ASO molecules are stabilized in the structure mainly via interactions of 2′-MOE groups of the first nucleotides with the protein molecules. 2′-MOE G1 of the first strand makes contacts with K204, and 2′-MOE G1 of the other strand interacts with R77 of the symmetry-related protein molecule, forming a crystal contact. Therefore, this part of the ASO is placed snugly between two protomers of AnxA2 in the crystal, which further stabilizes the crystal lattice (Supplementary Figure S2A). 2′-MOE groups are the largest 2′ modifications that were used in the crystallization trials. We hypothesize that the ability of 2′-MOE to form such extensive interactions with the protein surface facilitated formation of the crystals of an otherwise highly dynamic protein-ASO complex.

**Figure 2.**
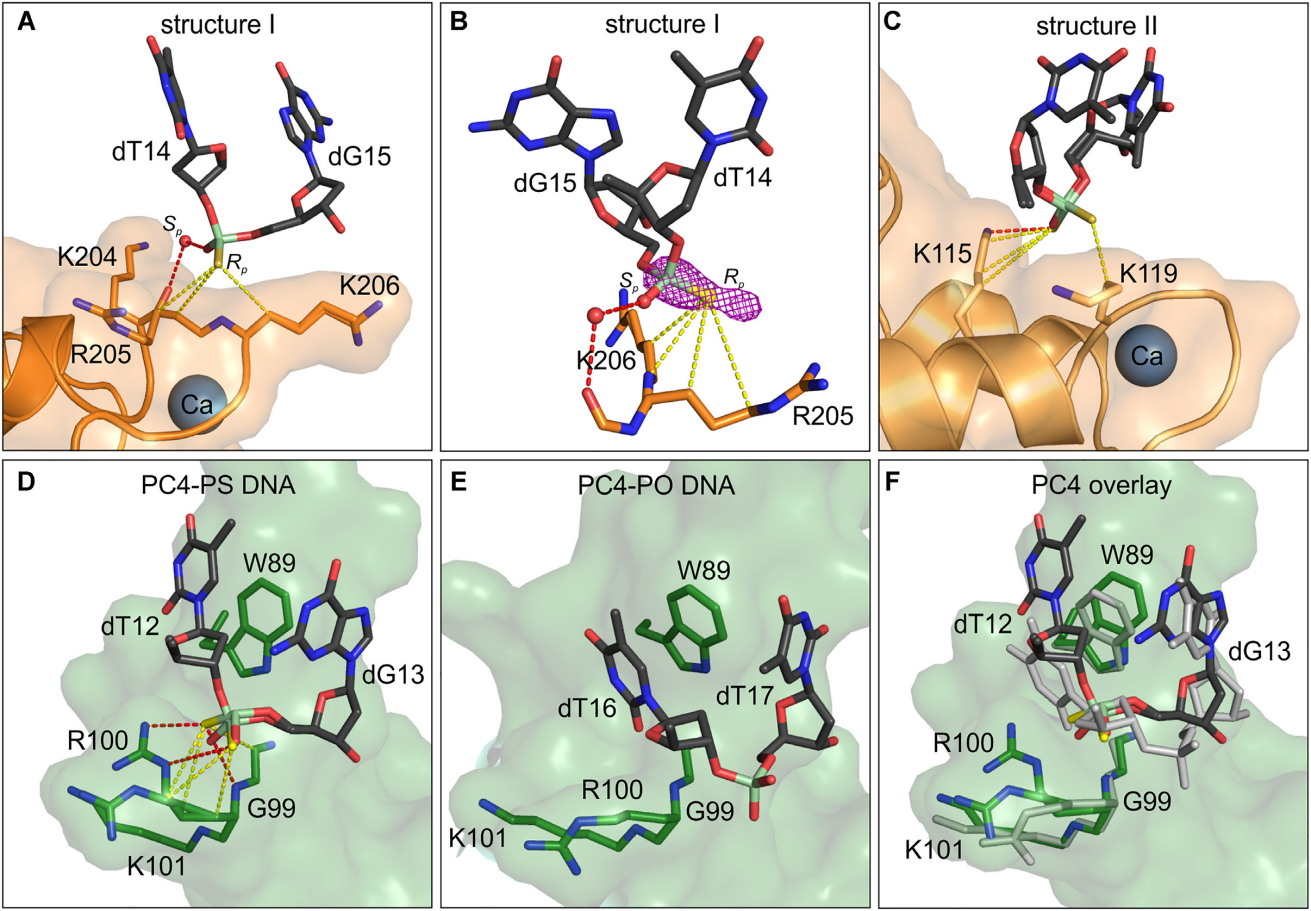
Phosphorothioate-binding pockets in AnxA2 and PC4. (**A**) Protein-PS ASO interactions in structure I. (**B**) Difference Fourier anomalous map calculated based on long-wavelength X-ray diffraction data (λ = 2.7552 Å) is shown for PS linkage from (A), contoured at 3.5 σ. (**C**) Protein-PS ASO interactions in structure II. (**D**) Protein-PS ASO interactions with PC4 (PDB ID: 6YCS). (**E**) Protein-PO DNA interactions with PC4 (PDB ID: 2C62). (**F**) comparison of PS ASO vs. PO DNA interactions at the nucleotide binding pocket in PC4. The proteins are shown in cartoon and transparent surface representation and colored orange for AnxA2 and green for PC4. Key residues that are involved in DNA binding are shown as sticks and labeled. The nucleotides are shown as dark gray sticks and labeled. The key amino acids and nucleotides from the PC4-PO DNA binding pocket are shown in (F) as gray sticks. For the stereorandom PS ASO model, the backbone is shown for both *R*p*/S*p diastereoisomers, and nucleosides are shown only for one diastereoisomer (*R*p PS) for clarity. Phosphorus atoms are shown in pale green. Oxygen atoms are shown in red. Nitrogen atoms are shown in blue. Sulfur atoms are shown in yellow. Polar interactions are shown as red dotted lines. van der Waals interactions are shown as yellow dotted lines. Ca^2+^ ions are shown as spheres.

Most of the ASO duplex does not contact the protein. The key interaction is formed between loop IIIAB (the convex surface of AnxA2) and the phosphorothioate between nucleotides dT14 and dG15 close to the 3′ end of the DNA (Figure 1C). This part of the ASO adopts a distorted conformation that allows the phosphorothioate linkage to interact with a pocket on the surface of the protein that is created by R205 and K206. The interaction is primarily mediated by contacts between the backbone and aliphatic parts of both amino acid side chains that make van der Waals contacts with the PS group (Figure 2A). Closer inspection of the binding site clearly demonstrates that the interactions with lysine and arginine residues in this structure would only be possible when the non-bridging sulfur is in the *R*p position of the PS stereoisomer. Only in this position is the sulfur atom at a distance that allows van der Waals contacts with the R205 and K206 side chains, whereas in the *S*p conformation, it would be too distant from these amino acids. An additional water-mediated contact is also made by the non-bridging oxygen atom with the backbone amide oxygen atom of K204. This indicates the preferential binding of one diastereoisomer at this site. To validate the assumed stereo configuration, we collected X-ray diffraction data at a wavelength of λ = 2.7552 Å to exploit the increased anomalous signal from sulfur atoms. Phases from the refined model of structure I were used to generate an anomalous difference Fourier map. Distinct electron density from the calculated difference map was clearly visible around the phosphorus atom and *R*p non-bridging atom in the inspected PS linkage (Figure 2B), which unambiguously confirmed that only this PS stereoisomer is bound at this site. This clearly indicates that it is the hydrophobic nature of the sulfur atom that contributes to nonspecific binding with AnxA2.

In structure II of the AnxA2–ASO complex, visible nucleotides were located in the vicinity of loop AB of domain II (Figure 1D). Notably, this loop is the equivalent of loop IIIAB, which binds the PS group in structure I. The PS linkage makes van der Waals contacts with side chains of K115 and K119 (Figure 2C). In contrast to the distorted trajectory in structure I, the oligonucleotide backbone that is bound by the protein in structure II adopts a more canonical conformation. In both complexes, however, the phosphorothioates that are bound by the proteins show similar interactions with hydrophobic portions of surrounding amino acids (Figure 2A, C). This mode of interaction is analogous to previously reported contacts of the PS backbone with lysine and arginine residues in the PC4-ASO complex (Figure 2D). For both AnxA2 and PC4, these two amino acids play a key role in forming unique interactions with PS groups. Interestingly, in the PC4-ASO structure, one of the phosphorothioate groups occupies a hydrophobic pocket that, for regular phosphodiester (PO) DNA, accommodates a nucleobase (Figure 2E, F). In PC4, the binding of PS leads to alterations of conformation of the side chain of R100 and the formation of extensive van der Waals contacts between the sulfur atom and aliphatic part of the amino acid. Therefore, the PS backbone can bind at pre-formed binding pockets on the protein surface and induce local conformational rearrangements of amino acid side chains to facilitate specific interactions. Overall, both AnxA2- and PC4-PS ASO structures demonstrate that unique contacts of the sulfur atom with amphipathic side chains of arginine and lysine play a key role in the enhanced affinity of PS ASO for proteins.

The distorted trajectory of PS ASO interacting with AnxA2 domain III is partially stabilized by crystal contact of the oligonucleotide with another AnxA2 protomer in the lattice (Supplementary Figure S2A). The 3′-end that comprises nucleotides 14 and 15 is positioned in a cleft between two symmetry-related molecules of AnxA2 and makes extensive polar contacts with the symmetry-related protomer (Supplementary Figure S2B). This observation implied the possibility of ASO-induced protein oligomerization. To test this, we performed size exclusion chromatography experiments, but they did not show any indication of AnxA2-ASO oligomerization or aggregation (Supplementary Figure S2C). This demonstrates that in solution, AnxA2–ASO complexes do not form higher-order assemblies.

### Phosphorothioate stereoisomer preference is driven by the hydrophobic environment around the PS linkage

In the context of therapeutic nucleic acids, the synthesis of PS ASOs is not stereoselective, and an n-mer oligonucleotide is a racemic mixture of 2^(n-1)^ discrete *R*p and *S*p stereoisomers. The way in which this affects ASO-protein interactions and whether certain stereoisomers bind preferentially to the protein surface has not been elucidated. To address this issue, we further analyzed the anomalous difference Fourier map that was calculated from the long-wavelength X-ray diffraction data (λ = 2.7552 Å) for structure I (Table 1). We reasoned that these maps would allow us to determine whether specific stereoisomers preferentially occur at particular positions of the ASO duplex in the crystal.

**Figure 3.**
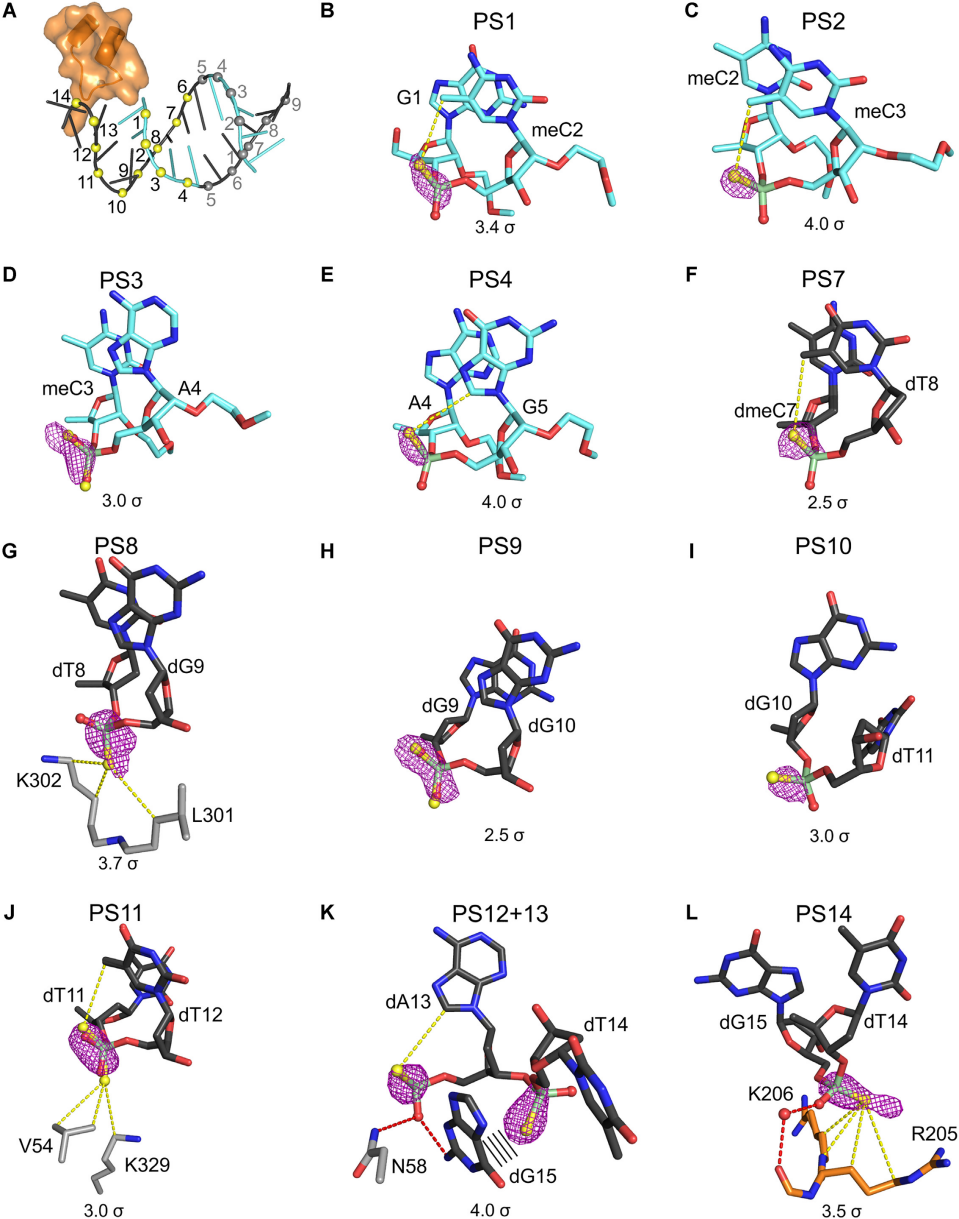
Phased anomalous difference Fourier maps for partial ASO duplex. (**A**) Schematic representation of partial ASO duplex in AnxA2-ASO structure I. The interacting part of AnxA2 is shown in cartoon and surface representation. Two ASO molecules are shown in cartoon representation. 2′-MOE PS nucleotides are shown in cyan, and PS DNA gapmer nucleotides are shown in black. PS linkages are shown as yellow spheres and numbered. PS linkages for which the signal was not observed in the anomalous difference maps are colored gray. (**B–L**) Difference Fourier anomalous maps calculated based on X-ray diffraction data collected at a wavelength of λ = 2.7552 Å are shown around PS linkages. Nucleotides and amino acids are shown as sticks and labeled. Phosphorus atoms are shown in pale green. Oxygen atoms are shown in red. Nitrogen atoms are shown in blue. Sulfur atoms are shown in yellow. The contour levels are indicated in each panel. The non-bridging sulfur and oxygen atoms are shown as yellow and red spheres, respectively. Polar interactions are shown as red dashed lines. van der Waals interactions are shown as yellow dashed lines. PS, phosphorothioate; meC, 5-methyl cytosine.

We obtained a high quality anomalous difference map with well-defined densities around the sulfur atoms. For PS linkages, clearly defined densities could be observed only for nucleotides that had B-factors below ∼60 Å^2^, namely for PS 1–4 for one ASO molecule and PS 6–14 for the second ASO molecule (Figure 3A). Careful inspection of the anomalous difference map revealed that at some PS linkages, one stereoisomer was preferentially bound; in other cases, the signal was distributed evenly between non-bridging atoms, indicating a lack of stereoisomer preference. Below we describe subsequent PS linkages starting from the 5′ end of the oligonucleotide. At PS1 (numbering from the 5′ end of the ASO; Figure 3A), the anomalous difference map clearly shows that the sulfur atom is in *R*p position (Figure 3B). The non-bridging sulfur atom in this position is located close to the methyl group from the m^5^C base, and a van der Waals interaction is possible. A similar situation is observed at PS2 (Figure 3C). Therefore, the presence of sulfur atoms in *R*p positions at these sites is presumably facilitated by the hydrophobic interaction with the m^5^C base (Supplementary Figure S3A, B). PS3 interlinkage shows no preferential occupancy of the sulfur atoms, with evenly distributed electron densities observed at both sides of the phosphorus atom (Figure 3D). No specific interactions were observed for non-bridging sulfur atoms at this site (Supplementary Figure S3C). In PS4, the sulfur atom is located in the *R*p position (Figure 3E). Here, a van der Waals contact is possible between the *R*p sulfur atom and the methylene group of the guanine base (Supplementary Figure S3D). The *R*p position is also observed for PS7 (Figure 3F). The sulfur atom is at a van der Waals distance from the methyl group from the thymine base (Supplementary Figure S3F). This is similar to the m^5^C contact near PS1 and PS2. In contrast, in the PS8 linkage, the sulfur is located in the *S*p position (Figure 3G). The non-bridging sulfur atom makes van der Waals contacts with hydrophobic portions of L301 and K302 from the symmetry-related AnxA2 molecule (Supplementary Figure S3G). The environment that surrounds PS9 is similar to PS3; accordingly, this phosphorothioate shows no stereospecificity (Figures 3H, S3H). At PS10, the sulfur atom is clearly located in the *R*p position, although no clear structural features could be observed that could explain this specificity (Figures 3I, S3I). For PS11, anomalous electron densities were evenly distributed around both non-bridging atoms of the phosphate, indicating that both *R*p and *S*p stereoisomers are present at this linkage (Figure 3J). The *R*p position is presumably facilitated by a van der Waals interaction with the methyl group of the thymine base, whereas the *S*p sulfur atom is stabilized by interactions with hydrophobic V54 and the ϵ-methylene group of K329 from the symmetry-related AnxA2 molecule in the crystal (Supplementary Figure S3J). The next two phosphates, PS12 and PS13, are stabilized by the guanine base from the last nucleotide of ASO (Figure 3K). At PS12, the *R*p sulfur atom is in proximity to the methylene group of the adenine base, whereas the *S*p oxygen makes polar contacts with the guanine base and N58 from the symmetry-related protein molecule (Supplementary Figure S3K). Conversely, PS13 is stabilized by T-shaped stacking with the same guanine base (Figure 3K). The last phosphorothioate, PS14, is the only phosphate that makes interactions with AnxA2 in the asymmetric unit of the crystal. Here, as described above, a distinct electron density from the anomalous difference map is clearly visible around the *R*p non-bridging atom, confirming that a stereoisomer with *R*p sulfur atom is bound to the protein at this site (Figures 3L, S3L). Altogether, these results indicate that stereoisomer preference at a given phosphorothioate in the crystal is driven by the hydrophobic environment around the PS linkage that is created by either the protein or surrounding portions of the nucleic acid.

### Phosphorothioate DNA is bound by lysine and arginine side chains in a calcium-dependent manner

**Figure 4.**
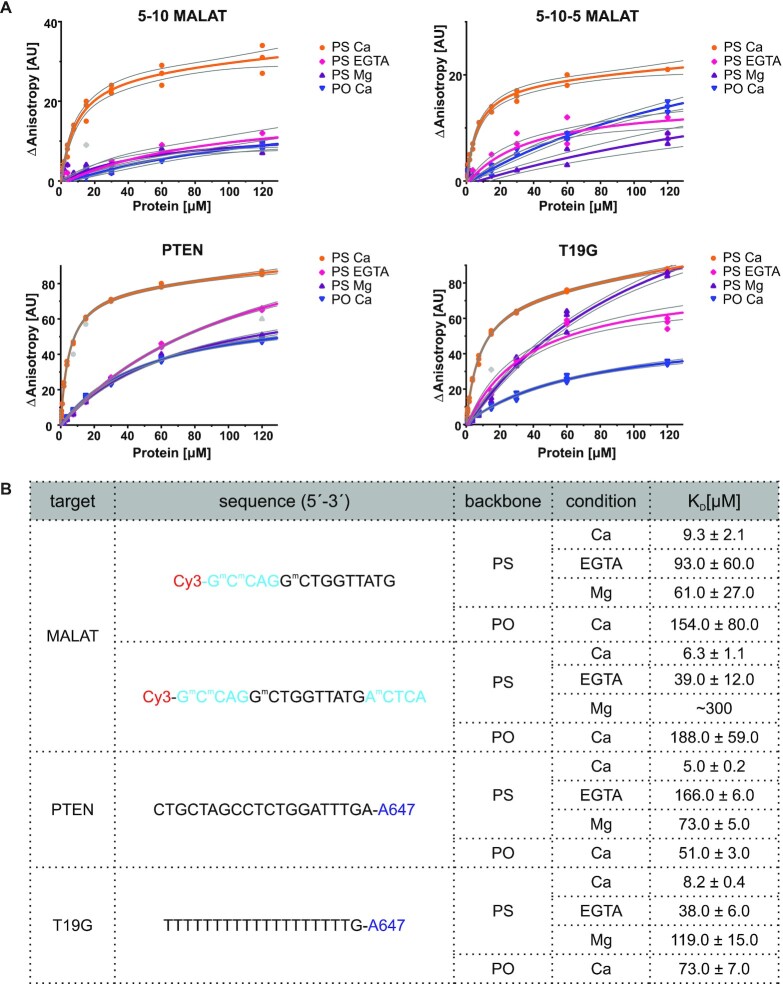
ASO binding by AnxA2 (fluorescence anisotropy titrations). Binding affinities were determined by incubating 50 nM fluorescently labeled DNA with AnxA2 at concentrations from 0.1 to 120 μM and measuring fluorescence anisotropy. The experiments were performed for the PO and PS version of each oligonucleotide in the presence of Ca^2+^, Mg^2+^ or EGTA. (**A**) Concentration–response curves. The ASO variants that were used in the study are indicated on the top of each plot. Thin gray lines border the 95% confidence bands for each fitted curve. All identified outliers were removed from the analysis and are indicated in gray. (**B**) Table of *K*_D_ values from fluorescence anisotropy measurements [μM]. Sequences of the oligonucleotides that were used in the binding assays are indicated. 2′-MOE wings are shown in cyan. DNA nucleotides are shown in black. The *K*_D_ values from fitting of the binding curves to the 1:1 binding equation (see Materials and Methods) ± standard error of the mean (SEM) are given. PS, phosphorothioate; PO, phosphodiester; ^m^C, 5-Me cytosine.

To further investigate the interaction between PS DNA and AnxA2, we performed binding assays using titrations with a fluorescence anisotropy readout. In these experiments, we used several fluorescently labeled oligonucleotides to establish the effect of backbone chemistry (PS versus PO), the presence of 2′-MOE wings, and the ASO sequence (Figure 4). Therapeutic ASOs are typically composed of a central stretch of a 10 nt DNA part (gapmer) that is flanked by short fragments of 2′-modified nucleotides (wings). Our crystal structures of AnxA2-ASO complexes contain a 15-mer oligonucleotide that lacks the 3′ wing (5–10 MALAT). To check whether the length of ASO affects the interaction with AnxA2, we used both the truncated (5–10) and full-length (5–10–5) versions of the MALAT ASO in our binding assays. In our experiments, we also included an ASO that targets human phosphatase and tensin homolog (*PTEN*) and lacks any 2′-modifications to test their effect and influence of altering the DNA sequence. Another DNA that was used was a model PS oligonucleotide with 19 thymidines and a guanosine that is unable to form stable secondary structures (T19G; Figure 4). For all oligonucleotides, we also included variants with a regular phosphate backbone (PO) as a reference. In our structures, the backbone of oligonucleotides is bound by protein loops that accommodate Ca^2+^ ions (Figure 2A, C). Thus, we reasoned that that the presence of Ca^2+^ ions might be required for efficient DNA binding. Therefore, we performed our assays in the presence of Ca^2+^, the presence of Mg^2+^, and without ions (i.e. in the presence of EGTA).

Fluorescence anisotropy binding assays showed that in the presence of Ca^2+^, all tested PS oligonucleotides bound to AnxA2 with similar affinities. The measured *K*_D_ values were 9.3 μM for 5–10 MALAT and 6.3 μM for 5–10–5 MALAT. This showed that increasing the length of the ASO led to a minor increase in AnxA2 affinity for DNA. The *K*_D_ values for PTEN ASO and T19G were also similar (5.0 and 8.2 μM, respectively). This indicated that the sequence of the DNA and presence of the 2′ modification have a small influence on affinity. This observation is in agreement with our crystal structures that show that the main interactions between the DNA and protein are mediated by the DNA backbone. Therefore, the sequence and sugar modifications should not significantly affect binding.

Notably, all four PO variants were bound by AnxA2 with significantly lower affinities (Figure 4). These results clearly demonstrate that PS linkages are of key importance for binding to AnxA2. This agrees with our structural data. In both AnxA2–ASO structures, the interactions between the PS linkage and protein side chains are mainly of hydrophobic character. These types of contacts are not created by regular phosphodiester linkages of natural nucleic acids, which explains the loss of affinity of PO oligonucleotides for AnxA2 in the binding assays.

Importantly, in the presence of EGTA, we observed decreased DNA binding for all oligonucleotide variants (from 5-fold decrease for T19G up to 23-fold decrease for PTEN), demonstrating that it is Ca^2+^-dependent. The observed effect was specific for Ca^2+^ ions because reduced binding was also detected in the presence of Mg^2+^ ions. Moreover, T19G PS DNA bound with affinity that was similar to MALAT oligonucleotides, indicating that the binding of ASO to AnxA2 in solution does not require ASO duplex formation. Hence, DNA duplex formation that was observed in structure I was promoted by crystal formation and is not required for AnxaA2-PS ASO binding.

**Figure 5.**
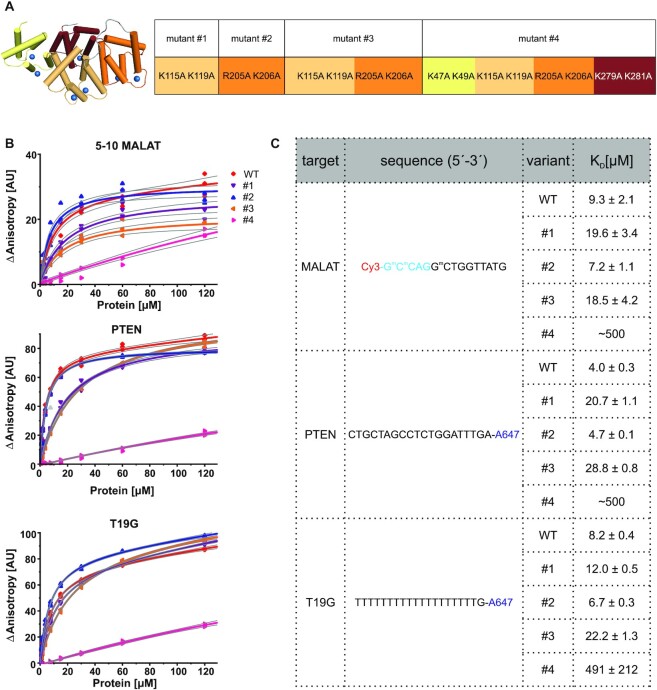
ASO binding by AnxA2 substitution variants. Binding affinities were determined by incubating 50 nM fluorescently labeled DNA with variants of AnxA2 at concentrations from 0.1 to 120 μM and measuring fluorescence anisotropy. The experiments were performed for the PS version of each oligonucleotide in the presence of Ca^2+^. (**A**) AnxA2 variants used in the experiments. The left panel shows the AnxA2 structure with annexin domains I-IV colored in yellow, pale orange, orange, and ruby, respectively. The right panel shows the design of mutated variants of AnxA2 that were used in the study. Sites of mutations are color-coded as in the left panel. (**B**) Concentration-response curves. The ASO variants that were used for the study are indicated on the top of each plot. Particular AnxA2 variants are color-coded as shown in the key. Thin gray lines border the 95% confidence bands for each fitted curve. All identified outliers were removed from the analysis and are indicated in gray. (**C**) Table of *K*_D_ values from fluorescence anisotropy measurements [μM]. Sequences of oligonucleotides that were used in the binding assays are indicated. 2′-MOE wings are shown in cyan. DNA nucleotides are shown in black. The *K*_D_ values from fitting of the binding curves to the 1:1 binding equation ± standard error of the mean (SEM) are given. ^m^C, 5-Me cytosine.

We next sought to validate the importance of contacts that were observed in crystal structures for AnxA2–ASO binding. We substituted residues that are involved in DNA backbone binding with alanines. We substituted amino acids that were observed to interact with PS DNA in structures I and II. This resulted in two protein variants: R205A/K206A (mutant #2) and K115A/K119A (mutant #1), respectively. We also prepared a variant in which all four residues were substituted with alanines (mutant #3) and a variant in which eight equivalent residues in all four annexin domains were mutated (mutant #4; Figure 5A). The recombinant proteins were purified, and their structural integrity was verified by Fourier-transform infrared spectroscopy. This analysis showed that all variants had the same content of secondary structures as the wildtype protein, indicating their proper folding (Supplementary Figure S4). The binding of proteins was tested for three different PS oligonucleotides: 5–10 MALAT, PTEN, and T19G. Overall, the mutations had a similar effect on binding for all tested ASOs (Figure 5B). Compared with wildtype protein, the variants with substitution in one or two annexin domains showed slightly lower affinity for PS ASOs. One exception was variant R205A/K206A (mutant #2), which had affinity for DNA that was comparable to wildtype protein. Notably, the weakening of affinity correlated with the number of mutated loops. When one binding site was disrupted (mutation variants #1 and #2), binding was moderately affected. The mutation of both binding sites that were detected in our crystal structures (mutant variant #3) further decreased affinity, with up to a ∼7-fold drop for PTEN ASO. Remarkably, mutations of all four binding sites resulted in the nearly complete loss of binding for all tested oligonucleotides. These results confirmed that the residues that were identified in our structures participate in PS DNA binding. Moreover, the lysine and arginine residues from the four calcium-binding loops play redundant roles in DNA binding, and only the substitutions in all repeated annexin domains completely abrogated binding.

## DISCUSSION

The phosphorothioate backbone is one of the most widely used modifications in therapeutic nucleic acids. Because of its remarkable nuclease stability and compatibility with the RNase H1-dependent mechanism of action, the incorporation of PS linkages in therapeutic oligonucleotides is currently the gold standard in the field (4,27–29). PS modification has been widely studied over the past 30 years, especially in terms of its chemical and pharmacological properties. Recent data on PS ASO interactions with cellular proteins revealed its profound consequences on the therapeutic index of these compounds. Despite this progress, the molecular mechanisms of ASO-protein interactions have not been fully established. We recently reported the co-crystal structure of a protein (transcription coactivator PC4) in complex with PS ASO, based on which we elucidated the structural basis for the higher affinity of phosphorothioate linkages to proteins (5). Here, we report a co-crystal structure of another model protein, AnxA2, in complex with PS ASO. We show that the interaction between PS linkages and lysine and arginine residues is a more general phenomenon that not only is observed for nucleic acid-binding proteins but may also account for associations between ASO and proteins that do not bind natural DNA.

In the present study, we describe extensive van der Waals contacts of the PS moiety with aliphatic parts of arginine and lysine side chains of AnxA2. The amphipathic nature of the sulfur atom has been proposed to enable PS linkages to form more dispersed, unique interactions with specific sites of proteins (27,30–34). The hydrophobic effect of the thiophosphate moiety on enhanced protein binding has been postulated in several studies, including studies of Antennapedia homeodomain (35), MS2-RNA hairpin (36), and SATB1-DNA (37) complexes. In the crystal structure of phosphorothioate-dependent restriction endonuclease ScoMcrA in complex with PS DNA, the phosphorothioate group is accommodated in a hydrophobic cavity that is formed by side chains of Tyr, Pro and Arg residues. Interestingly, this protein specifically recognizes and binds *R*p PS DNA. Only this configuration allows favorable hydrophobic interactions of the sulfur atom within the binding cavity.

A combination of electrostatic and hydrophobic contacts with side chains of amino acids was observed in the PC4-ASO complex (5). PC4 is a transcriptional coactivator with a canonical single-stranded DNA binding fold that can tightly bind both PO and PS oligonucleotides. In structural studies of PC4–ASO, we observed a mixture of canonical polar interactions between the DNA and binding domain that were enforced with hydrophobic contacts that were made by PS linkages. Furthermore, in the PC4–PS complex, presumably both PS stereoisomers were bound, creating a network of interactions at the binding pocket. The moderate resolution of the structure, however, did not allow the determination of stereoisomer preference. It is interesting to note that although in AnxA2–ASO structure the sulfur atom forms hydrophobic interactions with amino acid residues, these contacts are formed preferentially with methylene moieties of Arg and Lys and not with other hydrophobic residues. This is likely because the other hydrophobic amino acids are not exposed on the protein surface. However, it is also possible that the hydrated head groups of Arg and Lys play a role in ASO binding, perhaps in the initial approach of the nucleic acid to the protein.

AnxA2 does not contain any canonical nucleic acid binding motifs and did not bind PO oligonucleotides in solution. Therefore, the interactions that were observed in the AnxA2–ASO structure account for the binding of phosphorothioate DNA, independent of interactions with PO DNA, which is in contrast to the case of PC4. The AnxA2–ASO complex is thus a much less complex and more adequate model for analyses of molecular forces that drive the enhanced affinity of PS–ASO for proteins. Importantly, the high quality of the crystals allowed validation of the observed stereospecific interaction between the PS linkage and AnxA2 via the anomalous scattering of sulfur atoms. This unambiguously confirmed that van der Waals contacts between the sulfur atom and hydrophobic parts of the arginine and lysine side chains are the driving force for enhanced interactions between PS ASO and proteins. In principle, for racemic PS ASO we used for co-crystallization, the observed electron densities could arise from the averaged signal from a mixture of roughly 16 000 different combinations *R*p and *S*p PS linkages possible in a single 15 nt ASO strand. However, for some PS linkages we observed anomalous scattering indicating the presence of sulfur only for *R*p or *S*p atoms. It does not imply stereopurity of the crystallized oligonucleotide, but it indicates a significant enrichment of *R*p- or *S*p-PS moieties at certain positions in the ASO bound by AnxA2 in our crystal. This likely arises from a preference for particular stereoisomers of some PS linkages during complex and crystal formation. Interestingly, the results also revealed that stereoisomer preference at a given phosphorothioate in the crystal is driven by the hydrophobic environment around the PS linkage that is created either by the protein or surrounding portions of nucleic acid, including the methyl group of me^5^C. This might explain the effect of incorporating me^5^C into therapeutic oligonucleotides.

In AnxA2–ASO structures, direct interactions between the phosphorothioate and the protein surface are only observed for one PS linkage. Three additional protein-nucleic acid interactions mediated by PS linkages are observed in crystal contacts (between symmetry-related complexes in the crystal). The relatively small interaction interface in the crystal structure is in line with a non-specific character of the association of PS ASO with AnxA2. It is very likely that our structures represent certain binding modes out of many possible ones. These particular configurations may have been stabilized by crystal formation. This is also supported by the fact that we obtained two crystal forms of AnxA2-ASO complex and in each resulting structure the nucleic acid interacts with different parts of the protein (domain III in structure I and domain II in structure II). Nevertheless, capturing these interactions provides clear snapshots of non-specific protein-ASO interactions and explains how they could arise in a physiological context.

Collectively, our AnxA2–ASO structures, together with previously reported studies, support the general mechanism of the enhanced binding of PS nucleic acids to cellular proteins, which involves hydrophobic interactions of the sulfur atom with methyl, methylene, and aromatic side chains of surrounding amino acids. In cells, interactions between ASO and nonspecific binders might be quite dynamic and depend on availability of the binding pocket on the protein and conformation and elasticity of the oligonucleotide. The mode of binding might involve local rearrangements of amino acid conformations in preformed binding pockets to accommodate the phosphorothioate (as shown in the PC4–ASO complex). However, the presented AnxA2–ASO complex suggests that flexibility of the single-stranded oligonucleotide might also contribute to preferential, although nonspecific, binding of the PS linkage to hydrophobic patches on the protein surface.

Our structural data showed no direct interactions between Ca^2+^ ions and the PS backbone, but nucleic acid binding experiments clearly showed that the binding of PS ASO with AnxA2 depends on calcium. Reported interactions between annexins and anionic polymers, including phospholipids, heparin, and mRNA, also occur in a calcium-dependent manner, although the exact mechanism has not been fully elucidated. One possibility is that calcium might stabilize the conformation of the interacting loop as suggested by spectroscopic studies (38,39). However, crystallographic structures for AnxA2 without calcium that are available in RCSB PDB show no significant changes in loop conformations or amino acid arrangements compared with calcium-bound structures. Alternatively, the presence of positively charged Ca^2+^ ions at the binding site might alter the charge distribution around the loop region. The binding of Ca^2+^ ions could introduce a large positive charge that counteracts the negative surface charge that is created by coordinating oxygens from surrounding amino acids at the binding site. This in turn could facilitate the attraction of anionic polymers, including nucleic acids. After the ASO backbone is attracted to the loop site, the distance between the non-bridging sulfur of DNA and hydrophobic methylene groups of amino acids becomes sufficient to make van der Waals interactions. A similar mechanism was proposed by Swairjo and colleagues for annexin A5 binding to phospholipid membranes (40,41).

In our AnxA2–ASO structure, we observed a single protein molecule that bound to a partially double-stranded ASO duplex. This partial base pairing allows the formation of an ordered helical structure, contrary to otherwise flexible single-stranded oligonucleotide. We assume that the configuration that we observed in the crystal may not fully resemble the conformation of ASO in solution. However, we believe that duplex formation, together with further stabilization of the crystal lattice via stacking interactions of 2′-MOE wings with AnxA2 protomers, facilitated the generation of crystals and enabled obtaining structural information on preferred PS-protein interactions, which would not be possible for single-stranded ASO.

Single-stranded nucleic acids are highly flexible in solution and can adopt a wide range of different conformations (4,42). This property might play an important role for enhancing ASO affinity to cellular proteins. Gaus and colleagues demonstrated that the affinity of PS oligonucleotides for plasma proteins depends on flexibility of the molecule (43). Correspondingly, in pull-down experiments, AnxA2 is precipitated only with single-stranded ASO and not with ASO/RNA duplexes (12). Furthermore, single-stranded ASOs interact with more cellular proteins than ASO/RNA, including a number of non-nucleic acid binders. The double-stranded duplex that adopts a more rigid helical conformation might not be able to accommodate the thiophosphate at shallow hydrophobic patches on the protein surface, which is possible for single-stranded nucleic acids. The importance of backbone flexibility and its role in PS-protein interactions is demonstrated in our structure of the AnxA2–ASO complex, in which the interacting nucleotides adopt a distorted trajectory to make favorable contacts with the hydrophobic pocket that is created by lysine and arginine residues of AnxA2. Similar observation has been reported for mRNA 5′ cap analogs, which contain an O-to-S substitution within the β-phosphate. In their studies, Warminski and coworkers demonstrated that the accommodation of the substituted sulfur atom in a binding pocket on the surface of eIF4E is accompanied with significant conformational rearrangement of the 5′,5′-triphosphate chain, resulting in different positioning of the α-phosphate (44).

The enhanced affinity of PS linkages for cellular proteins allows the better retention, productive uptake, distribution, and endocytic trafficking of these therapeutic compounds (45). This property is, however, a double-edged sword. The nonspecific and undesirable binding of PS ASO to certain proteins might alter its localization and function, which can contribute to cytotoxic properties of the drug molecule (2,4). Therefore, extensive research is being conducted to achieve the most effective design of potent and safe antisense therapeutics. Several chemical strategies have been explored, based on the introduction of certain modifications to different elements of the nucleotides that comprise ASO (46). One recent design involves replacing PS linkages with mesylphosphoramidate (MsPA) moieties (47). This internucleotide linkage retains a negative charge on the phosphate backbone, although it lacks the amphiphatic sulfur atom. The substitution of only four PS linkages with MsPA in ASO gapmer significantly improved safety of the compound and decreased nucleolar protein mislocalization without compromising RNase H1-dependent cleavage. This aligns with our structural data, which indicate that the ability of the sulfur atom to form hydrophobic interactions with the protein surface accounts for the nonspecific binding of PS ASO to proteins. Nevertheless, PS remains one of the most useful modifications of therapeutic nucleic acids. Understanding the effects it exerts on the mechanism of action of ASOs is of key importance.

Significant progress has been made in recent years in the rational design of therapeutic ASOs. Structural studies provide valuable insights that can help improve the design of new-generation ASOs. The further identification of binding motifs and amino acids that are involved in interactions between PS nucleic acids and key cellular proteins will make important contributions to our understanding of the fate of ASOs in biological systems. When designed appropriately, these drug molecules can achieve ideal antisense potency with fewer adverse events to substantially increase safety and efficacy in patients.

## DATA AVAILABILITY

Data that support the findings of this study are available from the corresponding author upon reasonable request. The X-ray crystallographic data for AnxA2-PS ASO structures I and II were deposited in PDB (accession nos. 7ZVN and 7ZVX, respectively).

## Supplementary Material

gkac774_Supplemental_File
