# Supplementary material for: Structures of annexin A2-PS DNA complexes show dominance of hydrophobic interactions in phosphorothioate binding
Source: Nucleic Acids Res. 2022 Sep 17;51(3):1409–23. doi: 10.1093/nar/gkac774 (PMC9943651; doi:10.1093/nar/gkac774)
Supplement: gkac774_Supplemental_File [file gkac774_supplemental_file.pdf]

# Supplemental information

## Structures of annexin A2-PS DNA complexes show dominance of hydrophobic interactions in phosphorothioate binding

Malwina Hyjek-Składanowska, Brooke A. Anderson, Vitaliy Mykhaylyk, Christian Orr, Armin Wagner, Jarosław T. Poznański, Krzysztof Skowronek, Punit Seth and Marcin Nowotny

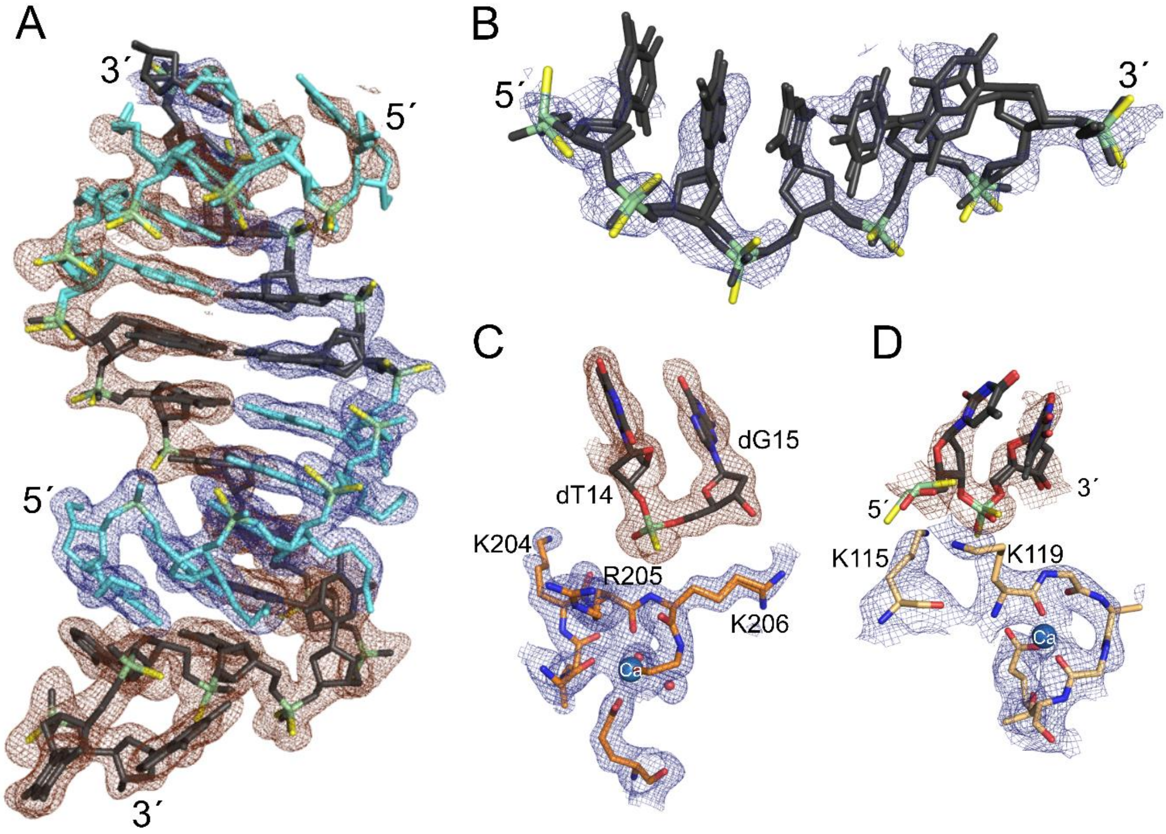


**Figure S1. Electron density maps.** (**A**, **B**) Simulated annealing composite omit electron density maps contoured at 1.0 σ overlaid on fragments around PS ASO in structures **I** (a) and **II** (b). For stereorandom PS linkages, the nucleic acid was modeled as alternative conformations of *pro-*$R_p$ and *pro-*$S_p$ PS diastereoisomers, and both conformations are shown. 2′-MOE PS nucleotides are shown as aquamarine sticks, and PS DNA nucleotides are shown as dark gray sticks. The phosphorus atoms are shown in pale green. Sulfur atoms are shown in yellow. The 2Fo-Fc simulated annealing composite-omit map is shown as blue and brown mesh for each copy of the oligonucleotide. (**C, D**) Simulated annealing composite omit electron density maps contoured at 1.0 σ overlaid on fragments of the structure around the PS ASO-AnxA2 interaction site in structures I (C) and II (D). Amino acids are shown as sticks and labeled. PS DNA nucleotides are shown as dark gray sticks and labeled. Oxygen atoms are shown in red. Nitrogen atoms are shown in blue. Phosphorus atoms are shown in pale green. Sulfur atoms are shown in yellow. Calcium ions are shown as blue spheres. Water molecules are shown as red spheres. The 2Fo-Fc simulated annealing composite-omit map is shown as blue mesh for the protein and brown mesh for the nucleic acid.

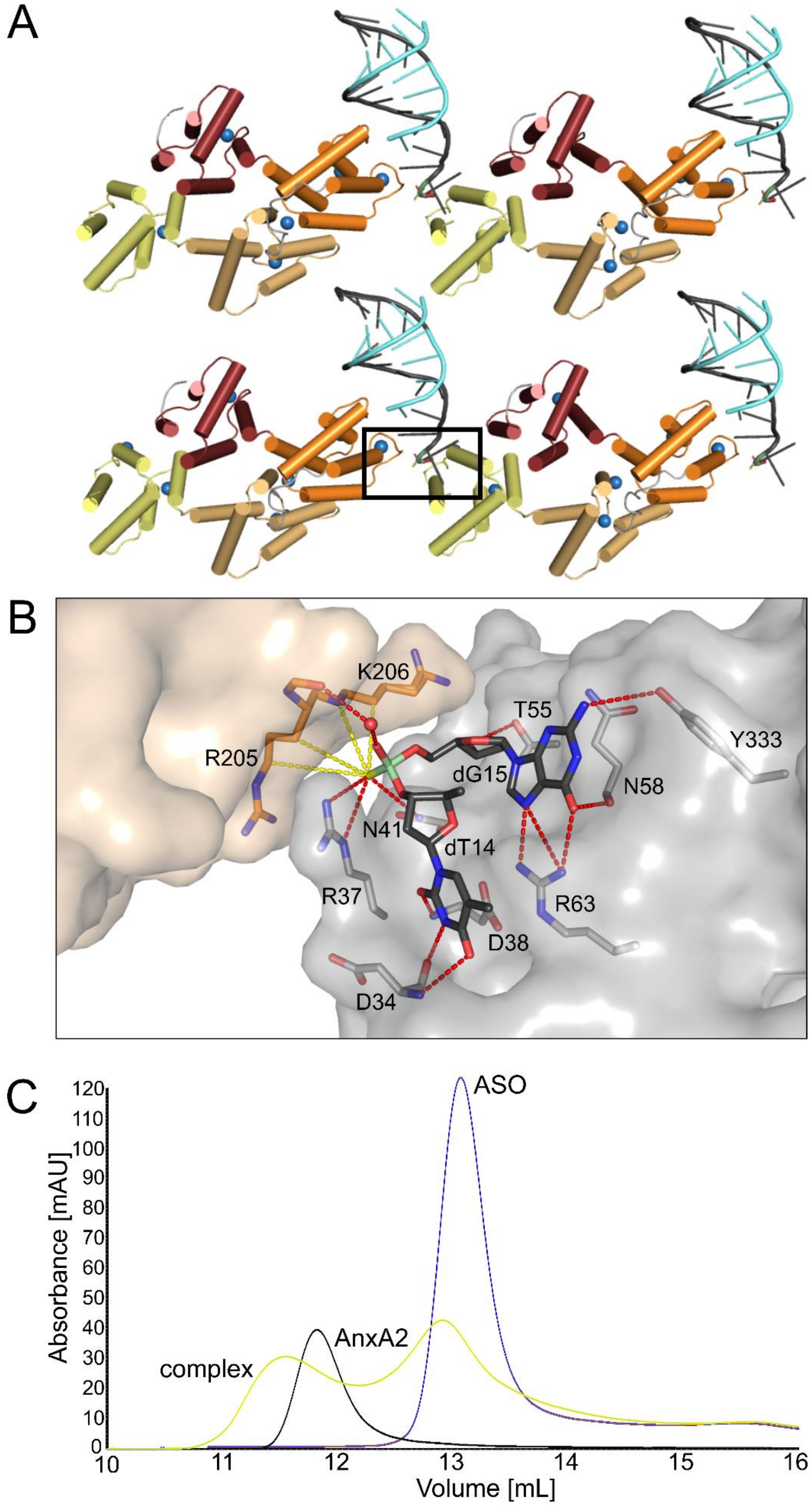


**Figure S2. Crystal packing of AnxA2 in complex with PS ASO in structure I.** (**A**) AnxA2 protomers

and PS ASO oligonucleotides are shown in cartoon representation. Calcium-binding annexin domains I-IV are colored yellow, pale orange, orange, and ruby, respectively. 2′-MOE nucleotides are colored cyan. DNA gapmer nucleotides are colored dark gray. Calcium ions are shown as blue spheres. The solid black box indicates the ASO-protein binding site, enlarged in (B). (**B**) Crystal contacts of PS linkage with symmetry-related AnxA2 molecules. The protomers are shown in cartoon and transparent surface representation and colored orange for AnxA2 from the unit cell and gray for the symmetry-related molecule. Key residues that are involved in DNA binding are shown as sticks and labeled. The nucleotides are shown as dark gray sticks and labeled. Phosphorus atoms are shown in pale green. Oxygen atoms are shown in red. Nitrogen atoms are shown in blue. Sulfur atoms are shown in yellow. Polar interactions are shown as red dotted lines. van der Waals interactions are shown as yellow dotted lines. The water molecule is shown as a red sphere. (**C**) Oligomeric state analysis of AnxA2 and AnxA2-PS ASO complex, determined using analytical gel filtration. Purified AnxA2, 5-10 PS 2′-MOE ASO, and AnxA2-ASO complex were applied to a Superdex 75 10/300 Increase size exclusion column in buffer that contained 20 mM HEPES (pH 7.0), 100 mM NaCl, 1 mM DTT, and 1 mM $CaCl_2$.

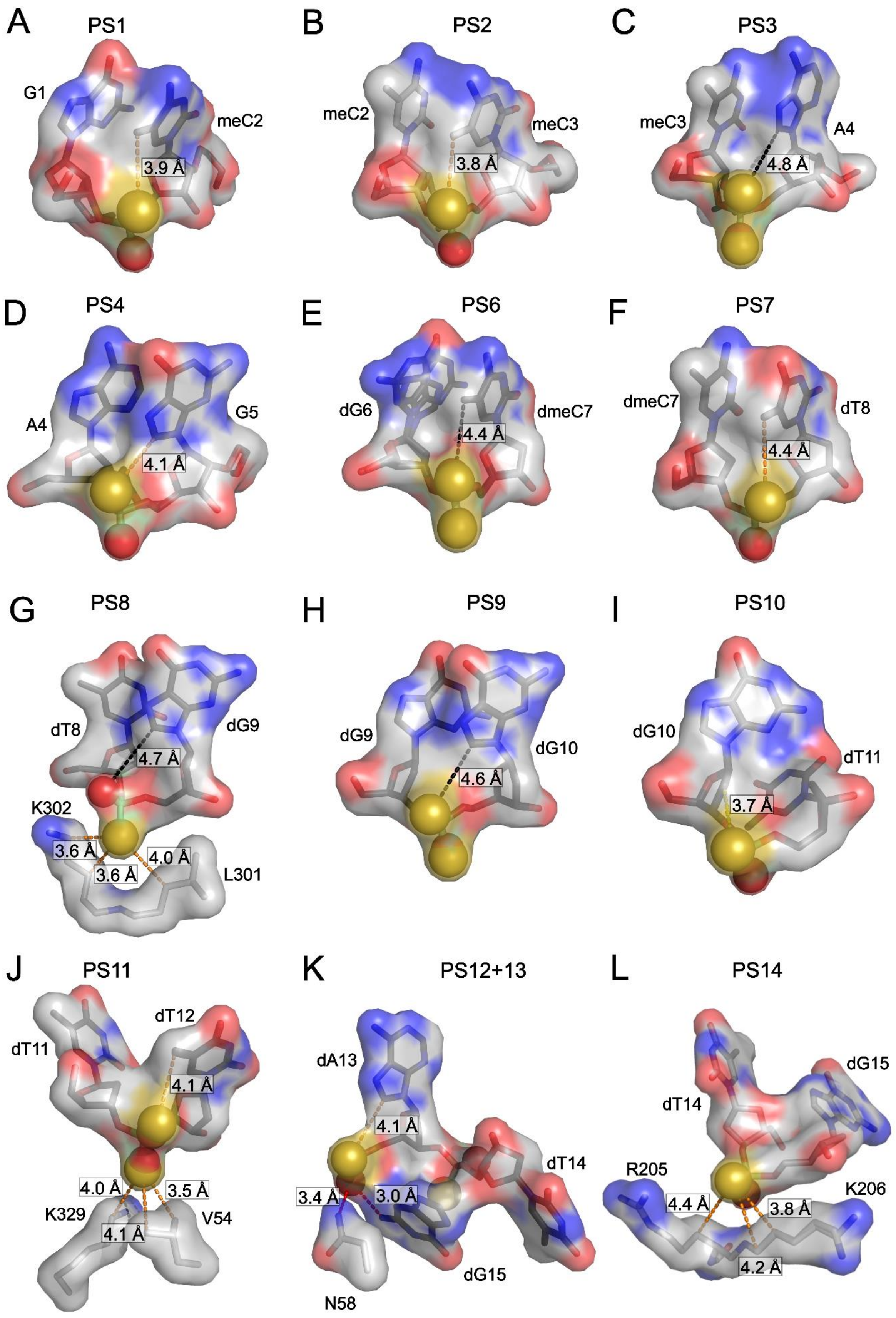


**Figure S3. Analysis of preferential stereoisomer occurrence throughout the ASO duplex in structure I.** Nucleotides and amino acids are shown as sticks and transparent surface representation

and labeled. Phosphorus atoms are shown in pale green. Oxygen atoms are shown in red. Nitrogen atoms are shown in blue. Sulfur atoms are shown in yellow. The non-bridging sulfur and oxygen atoms are shown as spheres. van der Waals contacts at a distance that allows for interactions are shown as orange dashed lines. van der Waals contacts at a distance that precludes interactions are shown as black dashed lines. PS, phosphorothioate; meC, 5-methyl cytosine.

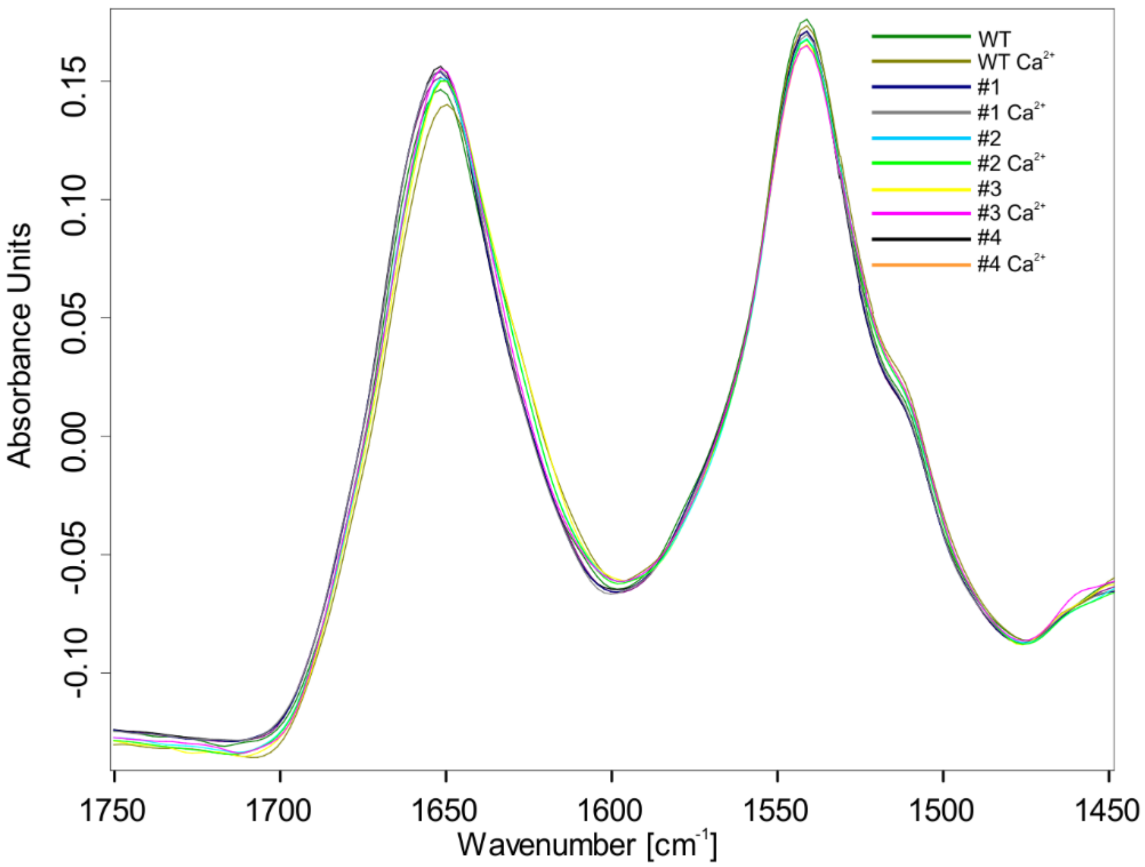


**Figure S4. Secondary structure content and structural integrity of AnxA2 variants.** Fourier-transform infrared spectrum of AnxA2 and four mutants in the presence and absence of $Ca^{2+}$ ions.
